# Proceedings of the Esculapian Society, Held in Charleston, Ill.

**Published:** 1857-08

**Authors:** F. R. Payne, D. W. Stormont

**Affiliations:** President; Secretary


### PROCEEDINGS OF MEDICAL SOCIETIES.

*Proceedings of the Esculapian Society, at the Semi-Annual Meeting, held in Charleston, Ill., May 27th and 28th,* 1857.

The Esculapian Society met in the Presbyterian Church, in Charleston, May 27th, and was called to order by the President, Dr. Payne, at 1 o'clock.

About twenty members were in attendance.

The minutes of the last meeting were read by the Secretary, and approved.

Dr. Chambers, chairman of the Committee on the Sale of Patent Nostrums, reported some reasons for not having discharged the duty assigned them. On motion of Dr. Steele, the committee were discharged.

The Treasurer stated that he was not ready to report on the condition of the finances of the society. On motion of Dr. Chambers, he was excused until the next meeting.

Dr. McCord was appointed to fill a vacancy in the Board of Censors.

The Secretary submitted the correspondence held with the Smithsonian Institution, in reference to procuring meteorological instruments for the use of the society. On motion of Dr. Chambers, it was laid on the table.

On motion of Dr. Chambers, Drs. Hoge, Brunk, Freeman and Morris, who were present, were invited to take seats, and participate in the discussions.

The following communication to the society, from Prof. Lawson, was presented by Dr. Chambers, and ordered to be spread on the minutes, viz.:

"CINCINNATI, May 4th, 1857.

"*To the President and Members of the Esculapian Society.*

"Being engaged in the collection of facts bearing on the subject of pulmonary consumption, I would respectfully ask the privilege of presenting the following interrogations to the members of your society, with the request that those who can furnish information will communicate it to me as early as may be convenient.

"1. The increase or diminution of pulmonary consumption within the last few years?

"2. The effects of locality in producing or retarding the disease, especially the influence of miasm?

"3. Has it been observed to follow and be influenced by attacks of fever, periodical or continued?

"4. Have cases been observed in which it was developed after a full attack of small-pox?

"5. The most approved mode of treatment. Have any new remedies been employed?

"Answers to the above questions, or other points of a similar character, will be most gratefully acknowledged.

"Very respectfully, L. M. LAWSON, M. D.,

"Professor of the Theory and Practice of Medicine in the Medical College of Ohio."

Dr. Davis was excused for not reporting on pneumonia, and continued on the same subject.

Dr. Frizell was excused for not reporting.

Dr. Stormont read a paper on Irregular Contractions of the Uterus, which was discussed at some length.

Dr. York was excused for not having an essay on Opthalmia, and continued.

Dr. Chambers read a very excellent and lengthy essay on Stomatitis Materna. After some discussion upon it, the Society adjourned to meet at 8 o'clock.

### EVENING SESSION.

The Society met according to adjournment, the President in the chair.

Dr. Davis being introduced, delivered an eloquent address to a large and attentive audience.

After the dismissal of the audience, the discussion on Dr. Chambers' paper was continued, with much interest, by several members.

Drs. York, Chambers and McCord were appointed a business committee, to report to-morrow morning.

On motion of Dr. Chambers, the Society adjourned to meet at 8 o'clock to-morrow morning.

The members and some invited guests then repaired to Bunnel's Hotel, where a sumptuous repast was in waiting, furnished by the Physicians of Coles County.

### MORNING SESSION—MAY 28.

The Society met as per adjournment, the President in the chair.

The Business Committee reported the following subjects, and the gentlemen whose names are opposite were appointed to report at the next meeting, viz.:

Epidemic Opthalmia—Dr. York.

Phlegmonous Erysipelas—Dr. Frizell.

Pneumonia—Dr. Davis.

Scurvy—Dr. McAlmont.

Irritation, Inflammation, Induration and Ulceration of the Cervix Uteri, with use of Speculum—Dr. Chambers.

Bites and Stings of Reptiles and Insects—Dr. Apperson.
Anesthetic Agents—Dr. Lodge.
Dropsical Effusions—Dr. Stormont.
Ovariotomy—Dr. Hinkle.
Puerperal Fever—Dr. Lee.
Milk Sickness—Dr. McCord.
Vaccination and Re-Vaccination—Dr. Van Dyke.
Tracheotomy and Laryngotomy—Dr. Albin.
Permanent Cure of Reducible Hernia—Dr. Smith.
Influence of Miasm in Modifying Disease—Dr. H. R. Payne.
Cure of Intermittent Fever—Dr. Bridges.
Scrofula—Dr. Washburn.
Rheumatism—Dr. Silverthorn.
Small Pox—Dr. Smith.
Typhoid Fever—Dr. Chambers.

Dr. Chambers announced the death of Dr. Duffield, a member of the Society, and moved that a committee be appointed to inquire into the facts of his having converted the shoulder into the vertex presentation, and report at the next meeting. Carried. Drs. Chambers, Hinkle and McCord were appointed. It was also moved, and carried, that the President, Dr. York, and Dr. Steele, be a committee to report resolutions of condolence.

Drs. Smith and Lodge were excused for not having essays.

Dr. Hinkle reported a very interesting case of Lithotomy. Discussed by several members.

On motion of Dr. Chambers, the thanks of the Society were tendered Dr. Davis for his excellent address last evening, and a copy requested for publication.

Dr. Payne read a very valuable report on Practical Medicine.

On motion, the Society then adjourned to meet at 1 o'clock.

AFTERNOON SESSION.

The Society met, the President in the chair.

Dr. York offered the following resolutions, which were unanimously adopted, viz.:

*Whereas*, It has pleased the Divine Being, in the dispensation of his providence, to remove from our midst, by death, our much beloved associate and brother, Dr. W. B. Duffield, one of our most aged and active members ; therefore,

*Resolved*, That we deeply regret the departure from the scenes of his labor, of his usefulness and his fame, of this noble votary of a noble profession ; and that we feel that the profession has lost one of its brightest ornaments, and society one of its most useful members.

*Resolved*, That we sincerely sympathize with his bereaved family in their irreparable loss, which we trust is his eternal gain.

*Resolved*, That the Secretary furnish Mrs. Duffield with a copy of these proceedings.

Dr. Payne's report was then taken up and discussed at considerable length.

Dr. Hinkle was appointed to deliver the next public address.

Paris was selected as the place for holding the next meeting.

Drs. York, Davis and Stormont were appointed the Committee of Arrangement.

Dr. York offered the following resolution, which was unanimously adopted, viz.:

*Resolved*, That we tender our thanks to the Presbyterian Church of this place for the use of their house on this occasion.

On motion of Dr. Stormont, the following resolution was unanimously adopted, viz.;

*Resolved*, That the thanks of this Society be most cordially tendered to the Physicians of Charleston, for the liberal hospitality extended by them to us during the present session.

The following gentlemen were admitted to membership during the meeting, viz.; Dr. G. W. Albin, Neoga, Ill.; Dr. J. H. Apperson, Fillmore, Ill.; Dr. D. P. Lee, Ashby, Ill., Thesis on Dysentery ; Dr. J. Van Dyke, Ashmore, Ill., Thesis on Blood-letting ; Dr. C. McAlmont, Charleston, Ill., Thesis on Hœmoptisis ; Dr. J. L. Silverthorn, Charleston, Ill., Thesis on Pneumonia.

A copy of the proceedings were ordered to be published in the North-Western Medical and Surgical Journal.

On motion, the Society adjourned to meet in Paris the last Wednesday in October.

F. R. PAYNE, *President.*

D. W. STORMONT, *Secretary.*

